# History and clinical epidemiology of *NF2*-related schwannomatosis

**DOI:** 10.1007/s10689-025-00504-5

**Published:** 2025-10-29

**Authors:** D. Gareth Evans, Jaishri O. Blakeley, Scott R. Plotkin

**Affiliations:** 1 Division of Evolution, Infection and Genomic Science, University of Manchester, Manchester, UK; 2 Manchester Centre for Genomic Medicine, St Mary’s Hospital, Manchester, UK; 3 Department of Neurology, The Johns Hopkins School of Medicine, Baltimore, MD USA; 4 Department of Neurology and Cancer Center, Massachusetts General Hospital, Boston, MA USA

**Keywords:** Vestibular schwannoma, Neurofibromatosis type 2, NF2, Pathogenic variant, Incidence, Prevalence, Mortality

## Abstract

*NF2*-related schwanomatosis (*NF2-*SWN) (previously Neurofibromatosis 2) as characterised by bilateral vestibular schwannomas (VS) was first described in 1822. However, due to the erroneous conflation of individuals with bilateral eighth nerve tumours with von Recklinghausen disease (currently Neurofibromatosis 1, NF1) in 1917 the literature was confusing for much of the 20th century. Even when the conditions were separated officially in 1987 (with separate localisation of the genes), *NF2-*SWN remained classified as a neurofibromatosis despite the tumours pathognomonic for NF1, neurofibromas, not being a feature of NF2-schwannomatosis. It is only in 2022 that *NF2-*SWN was correctly delineated as a schwannomatosis. The epidemiology of *NF2-*SWN has only been possible to delineate after the separation of *NF2-*SWN from the much more frequent nerve sheath predisposing tumour condition NF1. Two research groups have published on the birth prevalence and population prevalence of *NF2-*SWN in the UK and Finland. The most highly ascertained assessment of *NF2-*SWN cases from the Manchester region of England (population 4.8 million) gave a diagnostic prevalence of 1 in 50,500 and calculated birth prevalences of 1 in 27,956 respectively. However, an updated prevalence across England in 2024 (population 55 million) gave a prevalence of at least 1 in 58,000. *NF2-*SWN usually presents with bilateral vestibular schwannoma, but can present with meningioma or spinal tumour or ophthalmic features before a VS diagnosis or with a unilateral VS and other tumours and rarely with a unilateral VS alone. Molecular testing is now extremely helpful in confirming the diagnosis of mosaic (present in up to 50% of de novo cases) versus germline *NF2* and distinguishing from other tumour predisposition conditions especially in childhood or cases with less common presentation. This chapter summarises the clinical epidemiology of *NF2-*SWN differentiating the condition from the overlapping non *NF2-*SWN.

## History of *NF2-SWN*

When telling the history of *NF2*-related schwannomatosis (*NF2-*SWN), it is important to also say something about the history of acoustic neuroma (now more accurately termed vestibular schwannoma, VS). Eduard Sandifort, Professor of Anatomy at the University of Leiden [current spelling], is probably the first to describe a VS in 1777 [1]. *NF2-*SWN was probably first described by the Edinburgh surgeon Wishart in 1822. He described a man with numerous intracranial nerve related tumours including what Wishart described as bilateral seventh nerve neuromas, but on review by Worster-Drought in 1937 [3] was re-labelled as eighth nerve tumours (now more accurately called VS). However, following the elegant delineation of type 1 neurofibromatosis (NF1) by von Recklinghausen (1882) [4], publications of *NF2-*SWN patients around the turn of the twentieth century were conflated with von Recklinghausen’s disease (NF1). Probably the most responsible for the prolonged misclassification of NF1 and *NF2-*SWN together was none other than Harvey Cushing who in 1917 stated that bilateral ‘nervus acusticus’ (VS) tumours were part of von Recklinghausen disease [5].

Despite many reports that emphasised the lack of cutaneous manifestations in *NF2-*SWN patients with bilateral VS and particularly the paucity of skin tumours and café- au-lait patches in patients and families with bilateral vestibular schwannomas [3, 6, 7] case series continued to conflate NF1 and *NF2-*SWN as a single disorder [8]. Even the report of a very large family with mild manifestations and without cutaneous features in 1930 [7] did not lead to a distinction between NF1 and *NF2-*SWN, although subsequently this led to a labelling of this mild phenotype as ‘Gardner type’ as opposed to the severe ‘Wishart’ type [2, 9]. [yes] The clinical and genetic distinction between the two conditions was not fully recognised until the last three decades of the twentieth century. Gradually, the differences in clinical presentation from NF1, led to the labelling of *NF2-*SWN as bilateral acoustic or central neurofibromatosis based primarily on work at the National Institutes of Health (NIH) in Maryland USA [10, 11]. The final formal separation of NF1 and *NF2-*SWN came in 1987 with the localisation of separate genes. That year, the *NF1* gene was localised to chromosome 17q [12] using family linkage and the *NF2* gene to 22q using tumour analysis and family linkage [13]. Because of this and the overwhelming clinical distinction to implicate two distinct conditions [11], the NIH Consensus statement published that year [14] formally separated the conditions into NF1 and *NF2-*SWN. Any residual concern that VS was still a manifestation of NF1 has now been refuted by large population-based studies [15, 16]. Previous publication of VS in NF1 were therefore contaminated with *NF2-SWN* cases [8]. Despite the clear evidence that nerve sheath tumours in *NF2-*SWN were ‘schwannomas’ rather than ‘neurofibromas’ with many previous reports of neurofibromas being reclassified on pathology review [9], the name neurofibromatosis for *NF2-*SWN still remained in consensus statements [17, 18]. Further delineation of *NF2-*SWN was made possible after the cloning of the *NF1* gene in 1990 and the *NF2* gene in 1993 [19, 20].

For the 32 years after the *NF2* gene cloning (including identification of *SMARCB1* as a schwannomatosis gene in 2007) there has been no evidence that classical *NF2-*SWN with bilateral VS was caused by any gene other than *NF2* [21]. The identification of *LZTR1* in 2014 [22] as a cause of the related schwannomatosis condition (multiple schwannomas but a lack of meningiomas, VS and ependymoma) resulted in significant clinical overlap between *NF2*-SWN and *LZTR1-*SWN. Early reports made clear that *LZTR1* germline variants were associated with unilateral VS [23] in patients meeting existing Manchester criteria for *NF2-*SWN [9, 24, 25] by having at least two additional non-VS schwannomas. For patients with unilateral VS and other non-intradermal schwannomas, *LZTR1* variants must ideally be ruled out before confirming a diagnosis of *NF2-SWN* [26–28]. Although young patients (< 25 years) with unilateral VS can be caused by heterozygous pathogenic variants in *LZTR1* or *NF2* or mosaic variants in *NF2* [29], families with multiple instances of unilateral VS are almost certainly not related to either condition[30].

A consensus process involving multiple experts was eventually co-ordinated by the Children’s Tumor Foundation (New York, NY, USA) involving multiple iterations and a Delphi process to arrive at new diagnostic criteria for *NF2*-SWN published in 2023 embracing most of the original Manchester criteria (s 1) but updating for new ophthalmic features and genetic testing Table 1 [28]. Although an age cut off for bilateral VS was suggested in 2019 (Table 2) [27] as most cases of isolated bilateral VS in people > 70 years old are predicted to be chance occurrences [31], the consensus was to retain all those with bilateral VS as possible *NF2*-SWN to dissuade clinicians from not investigating older patients.

**Table 1:** Original Manchester diagnostic criteria for NF2 [9]

Bilateral vestibular schwannomas OR family history of NF2 PLUS	
1) Unilateral acoustic (VS) OR	
2) Any two of: meningioma, glioma, neurofibroma, schwannoma, posterior subcapsular lenticular opacities	
*Additional criteria*	
2) Also cerebral calcification	
Unilateral VS + any 2 of: Meningioma, glioma, neurofibroma, schwannoma, cataract, cerebral calcification	
Multiple meningioma (2 or more) + unilateral acoustic or any 2 of: glioma, neurofibroma, schwannoma, cataract, cerebral calcification

**Table 2:** Revised manchester Criteria for NF2 [27]

1. Bilateral vestibular schwannomas < *70* OR	
2. Family history AND unilateral VS < *70* OR	
3. Family history OR unilateral VS AND two of*:	
[check:]meningioma, cataract, ependymoma, retinal hamartoma, schwannoma, cerebral calcification	
*(if UVS + ≥ 2 schwannomas need negative LZTR1 test) + , OR*	
4. Multiple meningioma (2 or more) AND two of:	
unilateral VS, cataract, glioma, neurofibroma, schwannoma, cerebral calcification	
5. Constitutional pathogenic *NF2* gene variant in blood or identical in two tumours** + **	

^*^Any two of includes two of any tumour type such as schwannoma, + 2016 suggested revisions

## Clinical manifestations and diagnostic criteria

A limited description of clinical manifestations is reported here as a companion paper will explore this more thoroughly. *NF2-*SWN is an autosomal dominantly inherited condition predisposing affected individuals to the development of schwannomas, meningiomas and ependymomas as well as specific ophthalmologic manifestations (posterior subcapsular or cortical cataracts, epiretinal membranes). As mentioned, bilateral VS are considered pathognomonic for *NF2*-SWN, but there are also often additional schwannomas of the other cranial, spinal, and peripheral nerves (including intradermal); meningiomas (intracranial and paraspinal), and some low-grade CNS malignancies (predominantly spinal > intracranial ependymomas) [9, 28]. Three large clinical studies in the early 1990’s confirmed the clinical phenotype [9, 11, 32]. The predominance of intrasmedullary tumours being spinal ependymomas and absence of high-grade gliomas has now been confirmed [33, 34] leading to gliomas being dropped from the diagnostic criteria [28]. Another important feature is a muscle wasting neuropathy which can be a mono-neuropathy including cranial nerves (particularly facial) and a generalised neuropathy [9]. The Manchester diagnostic criteria for *NF2-*SWN [9] are shown in Table 3. The original NIH criteria were expanded to include patients without a family history of *NF2-*SWN who have multiple schwannomas and/ or meningiomas, but who have not yet developed bilateral vestibular schwannoma. The Manchester criteria were demonstrated to have increased sensitivity and no less specificity than the original NIH criteria [14]. Further improvements were implemented to exclude neurofibroma and glioma in 2019 [28]. The international consensus group rejected the age limit to bilateral VS although the mere presence of bilateral VS alone after age 70 years has a very low detection rate of *NF2* pathogenic variants in blood (~ 6%) [27, 28].

**Table 3:** New revised diagnostic criteria for *NF2-*related schwannomatosis

A diagnosis of *NF2*-related schwannomatosis (previously termed neurofibromatosis 2,NF2) can be made when an individual has one of the following:	
1.Bilateral vestibular schwannomas (VS)	
2.An identical *NF2* pathogenic variant in at least 2 anatomically distinct NF2-related tumors (schwannoma, meningioma, and/or ependymoma). (Note: if the variant allele fraction (VAF) in unaffected tissues such as blood is clearly < 50%, the diagnosis is mosaic *NF2*-related schwannomatosis)	
3.Either 2 major or 1 major and 2 minor criteria as described in the following:	
*Major criteria:*	
Unilateral VS	
First-degree relative other than sibling with *NF2*-related schwannomatosis	
2 or more meningiomas (Note: single meningioma qualifies as minor criteria)	
*NF2* pathogenic variant in an unaffected tissue such as blood (Note: if the VAF is clearly < 50%, the diagnosis is mosaic *NF2*-related schwannomatosis)	
*Minor criteria:*	
Can count > 1 of a type (eg, 2 distinct schwannomas would count as 2 minor criteria)	
Ependymoma, meningioma (Note: multiple meningiomas qualify as a major criteria), schwannoma (Note: if the major criterion is unilateral VS, at least 1 schwannoma must be dermal in location)	
Can count only once (eg, bilateral cortical cataracts count as a single minor criterion)	
Juvenile subcapsular or cortical cataract, retinal hamartoma, epiretinal membrane in a person aged < 40 years, meningioma	

*NF2-*SWN patients may present with cranial meningioma(s) or spinal tumour(s) many years before the appearance of VS [9). It is now estimated that at least 73% of these? patients harbor de novo pathogenic variants in the *NF2* gene [35].

Ophthalmic features are also prominent in *NF2-*SWN and represent a common presenting feature in children [37–39]. With careful specialist ophthalmic assessment, 60–80% of *NF2-*SWN cases have evidence of cataract [32, 36]. These are most often juvenile posterior sub-capsular lenticular opacities (in contrast to age-related senile cataracts) that uncommonly require surgery or affect vision. Nonetheless, childhood cortical wedge opacities may be present from near birth [9]. Optic nerve sheath meningiomas can cause visual loss in the first years of life (but can also present much later) and retinal hamartomas also rarely can affect vision. Ophthalmic features are a strong part of the new diagnostic criteria (Table 1) which now include epiretinal membranes diagnosed < 40 years [28]. In *NF2-*SWN patients with severe manifestations, the combination of deafness and visual loss can lead to severe problems with communication.

Cutaneous examination is useful in diagnosis particularly in children. However, cutaneous manifestations in *NF2-*SWN are much more subtle than NF1. About 70% of *NF2-*SWN patients have cutaneous schwannomas, but only 10% have 10 or more [9]. Cutaneous intradermal schwannomas can be the presenting feature in childhood [35, 40]

## Birth prevalence and population prevalence

The first assessment of prevalence published for *NF2-*SWN was part of the NIH consensus statement [17] which estimated birth prevalence of 1 in 50,000. However, it is not clear how that figure was derived. The first true population-based estimate was from North-West England around Manchester in 1992 [41]. This estimated that *NF2-*SWN had a birth prevalence of 1 in 33–40,000 by mapping years of birth from regional data onto birth cohort data. Because many *NF2-SWN* patients do not develop features of *NF2-*SWN until their 30’s or later- and many died young–the actual diagnostic point prevalence was only 1 in 200,000 in 1992 [41]. The estimate of population diagnostic prevalence in Manchester increased to 1 in 56,000 in 2010 [42] and to 1 in 50,500 in 2018 [43], reflecting improved early diagnosis and better survival. The most recent figure from 2024 for the whole UK population found a prevalence of 1 in 58,000 for England but this was 1 in 55,000 for the three most highly ascertained regions [36]. The annual incidence rate was estimated in 1992 at 1 per 2,355,000 representing about one new case per year for each Health Region in the UK [41] or 100 cases per year in the USA. Since establishment of the *NF2*-SWN national service in England there have been 97 new cases in 15 years (2010–24) in the Manchester region (population 5 million) representing an annual incidence rate of 1 in 773,000. People who have *NF2-*SWN (particularly non-mosaic cases) have reduced genetic fitness and this is more marked in males, who may delay having children until their disease has progressed [41]. This reduction of heterozygotes from parental transmission is counterbalanced by the high de novo rate now estimated at 73% [36]. An alternative approach to calculating the birth prevalence based on VS diagnosis rather than *NF2-*SWN clinical criteria in North-West England in 2005 estimated a birth incidence of 1 in 25,000 [44]. Although these multiple studies in the Manchester region of currently 5 million people are based on a highly ascertained region, the overall point prevalence of *NF2-*SWN in the whole UK (population 68 million) is a little lower probably due to reduced ascertainment. A 2018 study found 932 patients meeting *NF2-*SWN criteria alive on prevalence day giving a prevalence of 1 in 67,700 although this increased to 1 in 58,000 in 2024. Given the particularly high de novo rate and similar prevalence in most of England, it is likely that birth prevalence globally for *NF2-*SWN will be around 1 in 25–30,000.

The only other population-based estimates published are from Finland. The first study was performed over an 11 year period in the Helsinki area in Finland finding that 3% of people with schwannoma and 1% of those that had meningioma had *NF2-*SWN [45]. However, a further 2% of schwannoma patients and 4% of meningioma patients had multiple tumours without fulfilling diagnostic criteria for *NF2-*SWN. The authors estimated that birth prevalence of *NF2-*SWN was 1 in 87,410 but this was based on a population of only 1.7 million and diagnosis necessitated having a tumour removed, which excluded those who were untreated or treated only with radiotherapy. A more recent whole population study in Finland (5.6 million) assessed birth prevalence at 1 in 39 000 in 2015 [46].

## Genetic mosaicism in *NF2-*SWN

The current assessment of birth prevalence and population prevalence includes people who do not have an identifiable pathogenic variant in *NF2* on blood analysis. This is because although testing sensitivity is ~ 95% in the generations after an index de novo case, testing sensitivity reduces to as little as 37% in sporadic cases [47]. Of 1055 de novo patients meeting Manchester *NF2-*SWN criteria, only 387 (36.7%) had a non-mosaic, heterozygous pathogenic *NF2* variant identified, although another 8% had detectable variants at mosaic levels in blood using a test for copy number variants and next generation sequencing [47]. Using further work on testing of tumours, the current estimate for mosaicism in a de novo* NF2-*SWN case is close to 60% [47]. Thus, in ~ 60% of new onset non-familial cases, the pathogenic variant is not present at conception but occurs during embryogenesis leading to only a small proportion of affected cells with the pathogenic variant that may be confined to neural crest cells. Mosaic *NF2-*SWN frequently demonstrates asymmetry with tumours predominantly on one side or one body segment [47, 48] or can be indistinguishable from non-mosaic patients with bilateral disease. The first documented case of mosaicism in *NF2-*SWN dates back to 1995 [49] and multiple series have been published since [50, 51]. Given that as many of 60% of people with a clinical presentation consistent with *NF2*-SWN may have mosaic disease, clinicians should be aware of the need to test blood and ideally ≥ 2 potentially associated tumours (i.e. schwannoma, meningioma, ependymoma) from distinct regions of the nervous system. If a shared *NF2* variant is seen across the unique tumour samples and not found (or at low level) in blood, this confirms mosaic *NF2*-SWN. People with mosaic *NF2-*SWN may still have severe clinical course and require appropriate clinical surveillance and intervention, but there is an implication of lower risk of transmission to children versus germline involvement. Overall based on the most recent epidemiological study in th UK [36] roughly 25% of *NF2*-SWN patients alive had inherited the disease from an affected parent 30% were de novo heterozygotes and 45% mosaics.

## Differential diagnosis

The main differential diagnosis for *NF2-*SWN includes non-*NF2* related schwannomatosis which usually presents with multiple non-vestibular painful schwannomas [28]. Some affected individuals, particularly at younger ages, who met older criteria for schwannomatosis without a VS had a mosaic *NF2* pathogenic variant [27, 43, 52]. Groups of patients with tumours largely confined to the peripheral nerves and spine, with sparing of the eighth nerve usually do not to have an identifiable *NF2* pathogenic variant identifiable in blood [52]. Three genes centromeric to *NF2* on 22q have now been found shown to account for ~ 70% of familial and ~ 30% of sporadic patients with multiple non-vestibular schwannomas: *SMARCB1* [53], *LZTR1* [22], and much less commonly *DGCR8* [54]. Patients with unilateral vestibular schwannoma and other schwannomas may have an *LZTR1* heterozygote pathogenic variant [23, 26, 27]. The important feature to distinguish non-*NF2*-related schwannomatosis is the absence of other *NF2*-SWN-related tumours such as intradermal schwannoma and ependymoma or ophthalmic features [27, 28]. Although inclusion of *LZTR1-*SWN could erroneously inflate the birth prevalence estimates for clinical *NF2-*SWN these rates have been adjusted for in the recent estimates [36, 43] and may not account for the 50% of new onset schwannomatosis cases without identifiable germline *SMARCB1* or *LZTR1* variants that on analysing ≥ 2 tumours have mosaic *NF2-*SWN [27, 36].

Outside of genetic tumour predisposition conditions, conditions that involve cranial nerve deposits such as leptomeningeal carcinomatosis, chronic meningitis (i.e. tuberculosis, syphilis, sarcoidosis) can mimic schwannomas involving multiple cranial nerves. In these cases there is often a clinical history that indicates a more rapid clinical course, elevated intracranial pressure and involvement of intracranial regions atypical for the tumours of *NF2-*SWN (i.e. basal cisterns). In the setting of multiple meningiomas, although *NF2*-SWN is the most common tumour predisposition condition for people with ≥ 2 meningiomas before age 40, peripheral schwannomas and intracranial meningiomas can be seen in the chromosome 22 condition *SMARCB1*-SWN. In the absence of schwannomas, there are additional genetic associations with multiple meningiomas such as *BAP1* tumour predisposition syndrome and *SUFU* familial meningioma predisposition as well as familial clear cell meningioma caused by *SMARCE1* and these can be assessed via commercially available multiple meningioma next generation sequencing panels. Finally, past medical history should be interrogated for prior radiation exposure and a common secondary neoplasm after remote radiation exposure is meningioma.

## Survival and mortality

Prior to 1990, survival for *NF2-*SWN patients averaged only 15 years post clinical diagnosis with a mean actuarial survival of ~ 60 years [9]. Many patients with severe disease were not surviving beyond their twenties and thirties. This life expectancy has improved in the last 30 years but overall, *NF2-*SWN patients still had significantly reduced survival compared to the general population in 2012 [55]. The improvement in survival based on treatment at a specialist centre with more judicious use of surgery/radiation treatments (deferring aggressive surgeries) [56] led to the designation of four treatment centres to manage all *NF2-*SWN patients in England commissioned in 2009 resulting in further improvement in survival [57]. By integrating population-based studies with comprehensive genetic testing, researchers have identified important correlations between genotype and survival. On average, patients with constitutional truncating mutations have the worst overall survival while those with mosaic disease and those with missense variants have the best overall course [55–57]. Those with splice-site or missense mutations had significantly lower mortality than patients with truncating variants (Odds ratio (OR) = 0.459, 95% CI 0.21–0.990, and OR-0.196, 95% CI 0.213–0.990, respectively), and those with splice-site variants in exons 6–15 had lower mortality than patients with splice-site mutations in exons 1–5 (OR 0.333, 95% CI 0.129 to 0.858) [56]. A revised score of genetic severity also recognises that truncating variants in exons 1 and 14–15 are associated with less severe disease than exons 2–13 [58]. Until recently the great majority of *NF2-*SWN patients died from complications of their disease [55, 57]. This can be related to multiple tumour progression with often the patient or their neurosurgeon deciding against further treatment. Loss of lower cranial nerve function can lead to aspiration pneumonia which is a common final diagnosis.

## New therapies

Additionally, although not yet directly assessed, increased use of systemic therapies may be improving survival in *NF2*-SWN. Specifically, in the setting of *NF2*-SWN the often large sizes and multiplicity of schwannomas and meningiomas and the cumulative toxicity of repeating local therapies such as surgery and stereotactic radiosurgery (SRS) has led to investigation of drug therapies for *NF2*-SWN tumors[59]. Therapies tested to date inhibit specific targets identified in preclinical and translational studies including VEGF, mTOR, EGFR/ErbB, ALK and FAK inhibitors [60–63]. In particular use of the VEGF antibody bevacizumab in the UK has shown particular efficacy and may have been part of the improved survival seen recently [56, 64, 65]. Some of these agents have shown significant activity against *NF2*-driven schwannoma and meningioma. However, none have been approved by regulatory bodies for the treatment of *NF2* driven tumors and are therefore used off label but with endorsement of national health agencies [64, 66].

## Conclusions

*NF2-*SWN has had a long history of conflation with the more common hereditary nerve sheath predisposing syndrome NF1 in the literature and in clinical practice. We now have robust diagnostic criteria which shows that *NF2-*SWN has a genetic cause, clinical course and outcomes completely unique from NF1. This is critical to recognize as there are unique approaches to diagnosis and management for *NF2*-SWN and it’s proximal conditions (such as *LZTR1*-SWN and *SMARCB1*-SWN). *NF2*-SWN has a birth prevalence globally estimated across multiple studies at 1 in 25–30,000. Because of diagnostics delays (especially in isolated cases) and reduced survival, the population diagnostic point prevalence even in populations with high ascertainment is closer to 1 in 50–60,000. Due to the high de novo pathogenic variant rate, poor genetic fitness of heterozygotes, and frequency of mosaic *NF2-*SWN, it is unlikely there are populations with substantially lower or higher rates. *NF2-*SWN is associated with reduced life expectancy although this has shown improvement with specialist care and new treatments such as bevacizumab. Survival is correlated with the type of underlying genetic variant with those harbouring heterozygous germline truncating variants in exons 2–13 having the worst survival.

## Data Availability

No datasets were generated or analysed during the current study.
